# Novel Dimer Derivatives of PF-543 as Potential Antitumor Agents for the Treatment of Non-Small Cell Lung Cancer

**DOI:** 10.3390/pharmaceutics14102035

**Published:** 2022-09-24

**Authors:** Su Bin Kim, Khem Raj Limbu, Yoon Sin Oh, Soo Lim Kim, Seung Ki Park, Dong Jae Baek, Eun-Young Park

**Affiliations:** 1 College of Pharmacy, Mokpo National University, Jeonnam 58554, Korea; 2 Department of Food and Nutrition, Eulji University, Seongnam 13135, Korea; 3 Department of Bio and Chemical Industry, College of Engineering, The University of Suwon, Hwaseong 18323, Korea

**Keywords:** non-small cell lung cancer, PF543, derivative, sphingosine kinase, anticancer

## Abstract

Lung cancer can be divided into non-small cell lung cancer (NSCLC) and small cell lung cancer, and the incidence and mortality rate are continuously increasing. In many cases, lung cancer cannot be completely treated with surgery, so chemotherapy is used in parallel; however, the treatment often fails due to drug resistance. Therefore, it is necessary to develop a new therapeutic agent with a new target. The expression of sphingosine kinase promotes cancer cell growth and survival and induces resistance to chemotherapeutic agents. Sphingosine-1-phosphate (S1P), produced by sphingosine kinase (SK), has been shown to regulate cancer cell death and proliferation. PF-543, currently known as an SK inhibitor, has been reported to demonstrate low anticancer activity in several cancers. Therefore, in this study, a derivative of PF-543 capable of increasing anticancer activity was synthesized and its efficacy was evaluated by using an NSCLC cell line and xenograft animal model. Based on the cytotoxic activity of the synthesized compound on lung cancer cells, the piperidine forms (Compounds **2** and **4**) were observed to exhibit superior anticancer activity than the pyrrolidine forms of the head group (Compounds **1** and **3**). Compounds **2** and **4** showed inhibitory effects on SK1 and SK2 activity, and S1P produced by SK was reduced by both compounds. Compounds **2** and **4** demonstrated an increase in the cytotoxicity in the NSCLC cells through increased apoptosis. As a result of using an SK1 and SK2 siRNA model to determine whether the cytotoxic effects of Compounds **2** and **4** were due to SK1 and SK2 inhibition, it was found that the cytotoxic effect of the derivative was SK1 and SK2 dependent. The metabolic stability of Compounds **2** and **4** was superior compared to PF-543, and the xenograft experiment was performed using Compound **4**, which had more excellent MS. Compound **4** demonstrated the inhibition of tumor formation. The results of this experiment suggest that the bulky tail structure of PF-543 derivatives is effective for mediating anticancer activity, and the results are expected to be applied to the treatment of NSCLC.

## 1. Introduction

Sphingolipids have been studied in various areas such as biology and biochemistry for a long time. They not only function in mediating the signal between cells but also regulate various biological reactions in small amounts. The structure of sphingolipids is very diverse and the mechanism of action in vivo is very complex. Sphingolipids are enzymatically transformed into various forms and each form plays a unique role. The ceramides also have different structures and roles depending on the type of fatty acid, which further complicates the role of sphingolipids. Increased levels of ceramide induce apoptosis. Sphingosine is changed to sphingosine-1-phosphate (S1P) by sphingosine kinase (SK), which further induces cell growth [1]. Various studies have demonstrated that S1P is related to the growth of cancer cells, and in fact, an abnormal increase in S1P levels has been observed in cancer patients [2,3]. Various SK inhibitors have been developed that can inhibit the growth of cancer cells through the regulation of SK. The developed SK inhibitors have been reported to effectively inhibit a wide variety of cancers in animal experiments [4]. SK has isotypes of SK1 and SK2, both of which have been reported to involve the growth of cancer cells, and are known to act at different locations within the cell [5]. However, it is currently unclear how to regulate the selectivity of SK1/2 through SK inhibitors and whether such selectivity is effective for anticancer effects.

**Figure 1:**
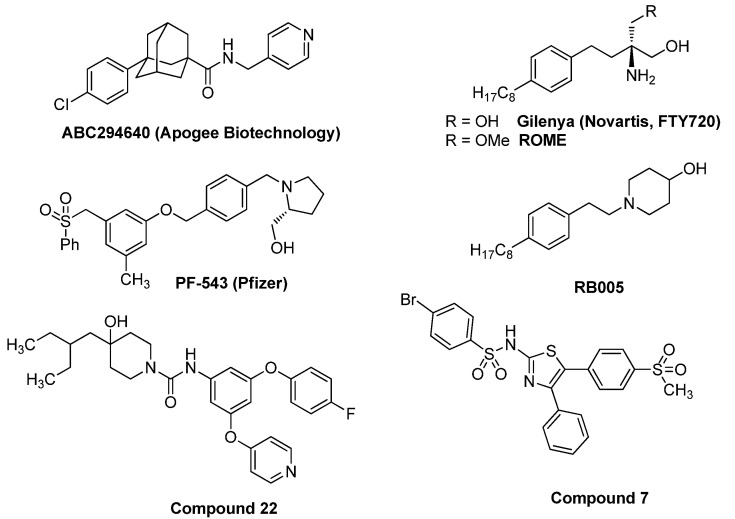
Known sphingosine kinase inhibitors.

Gilenya, known as FTY720 (Fingolimod) (Figure 1), works as a modulator of the S1P receptor and is a drug approved in 2010 for the treatment of multiple sclerosis [6]. FTY720 also selectively inhibits SK1 and is widely used as a parental structure of SK inhibitors. RB005 is an analog of FTY720 that selectively inhibits SK1, whereas (*R*)-FTY720 methyl ether (ROME) selectively inhibits SK2 (Figure 1), thereby showing a large change in selectivity for SK1/2 depending on the structure of the head group [7,8]. ABC294640 (Opaganib, Yeliva^®^ RedHill Biopharma), a nonlipid structure (Figure 1), selectively inhibits SK2 and is undergoing clinical trials in several types of cancer, including osteosarcoma [9]. However, not many inhibitors that selectively inhibit SK2 have been reported. PF-543, developed by Pfizer, has been reported to show the strongest SK1 inhibitory effect to date (Figure 1) [10]. Based on the results of PF-543 derivatives reported by other research groups, it has been identified that PF-543 still exhibits the strongest SK1 inhibitory effect. However, PF-543 had low anticancer activity in some cancers, and this effect seems to be the outcome of sustaining high sphingosine levels even under the suppression of S1P. Although the ultimate purpose of many reported SK inhibitors is to develop new anticancer drugs, low anticancer activity is one of the reasons for the persistence of SK inhibitors in the preclinical stage. PF-543 has demonstrated anticancer activity in colorectal cancer animal experiments, but unlike the other reported inhibitors, the anticancer activity of PF-543 was not high compared to its strong SK1 inhibitory effect [11]. Moreover, the low metabolic stability (MS) of PF-543 is one of the problems that needs to be addressed. Accordingly, the structural modification of PF-543 is required for high anticancer activity and stability, but there are not many derivatives reported so far [4].

When the methyl of the tolyl structure of PF-543 was removed, no consequences on the inhibitory effect, stability, and improvement of the anticancer activity of SK1 were observed [12]. On the contrary, in the case of the PF-543 analogs with a phenol backbone, the SK1 inhibitory effect and the anticancer activity were lowered, indicating that the nonpolar backbone should be retained [12]. According to a report of novel PF-543 analogues of Pfizer, there is no significant change in the SK1-inhibiting effect of analogues with the head group of the cyclic amine modified [10]. In 2019, the Pyne group reported a study showing that the selectivity of the SK1/2 inhibitory effect can be controlled through a small structural change in the benzenesulfonyl tail of PF-543 [13]. These results show that the tail structure of the inhibitor is important for the SK1/2 inhibitory effect and selectivity. Compound **22** (Figure 1), reported by Ono Pharma UK Ltd. of the UK in 2015, effectively inhibited S1P receptor 2 despite the presence of a bulky tail structure (Figure 1) [14]. According to recent research results, Compound **7** (Figure 1), which has a bulky structure containing a sulfonyl group, showed a high binding affinity for SK1 (K_a_ = 3.9 × 10^7^ M^−1^) and a high SK1 inhibitory effect (IC_50_ = 0.14 μM) [15]. Through the docking study, the researchers showed that the phenyl group of Compound **7** can interact with Phe173, Leu299, and Leu200 in SK1, which are the sites of interaction for the tolyl group of PF-543. These findings show that even bulky compounds can modulate sphingolipids such as SK1/2 or SIP receptors. Therefore, in our experiment, we studied whether the bulky tail group of the PF-543 analog had an effect on the SK inhibitory effect and whether it had an anticancer effect on non-small cell lung cancer (NSCLC) through SK.

## 2. Materials and Methods

### 2.1. Chemicals and Reagents

Roswell Park Memorial Institute-1640 (RPMI-1640), trypsin, and penicillin/streptomycin (P/S) were supplied from GE Healthcare Life Sciences Hyclone Laboratories (Pittsburg, PA, USA). Fetal bovine serum (FBS) was purchased from Gibco (Thermo Fisher Scientific Inc., Waltham, MA, USA). ApoScan^TM^ annexin V-FITC was obtained from BioBud (Seongnam, Gyeonggi-do, Korea). Cell viability assay kit EZ-CYTOX was received from Do-GenBio Co. Ltd. (Seoul, Korea). Antibodies against Caspase-3 (9662), BAX (2772), PARP (9542), and Bcl-2 (3498) were provided by Cell Signaling Technology (Danvers, MA, USA). β-actin (sc-47778) and HRP-conjugated anti-mouse and anti-rabbit antibodies were supplied from Santa Cruz Biotechnology (Dallas, TX, USA). SK1 (ab71700) and SK2 (ab264042) antibodies were acquired from Abcam (Cambridge, UK). Protein marker, protease, and phosphatase inhibitor cocktail were received from Thermo Fisher Scientific (Waltham, MA, USA). Electrochemiluminescence Solution for Western blotting imaging was offered by Millipore Corporation (Burlington, MA, USA). Alanine transaminase (ALT), aspartate aminotransferase (AST), and alkaline phosphatase (ALP) kits were purchased from Asan Corporation (Seoul, Korea). 

### 2.2. Cell Culture

The human lung adenocarcinoma cell lines A549 and H1299 were obtained from the Korea Cell Line Bank (Seoul, Korea). A549 and H1299 cells were maintained in RPMI-1640 culture medium supplemented with 10% FBS and 1% P/S in an incubator gassed with 5% CO_2_ at 37 °C. When cell confluence reached 80%, cells were passaged. 

### 2.3. Cell Proliferation Assay

The cell viability was evaluated by an EZ-CYTOX assay kit. Briefly, A549 and H1299 cells were plated in 96-well seeded at a density of 3 × 10^3^ cells/well overnight and then Compounds **1**–**4**, PF-543 and FTY720 were treated at different concentrations for 24 h, 48 h, or 72 h, respectively. Then, each well was treated with reagents from the EZ-CYTOX kit for 1 h. Finally, the cell viability was performed by measuring the absorbance with Multiskan go (Thermo Fisher Scientific, Waltham, MA, USA) at the 450 nm wavelength. 

### 2.4. Colony Formation Assay

A549 cells (1 × 10^3^ cells/well) were seeded into six-well plates and then each compound was treated at 5 and 10 μM for 12 h. The medium was changed every two days, and the cells were cultured in a fresh medium for 10 days. The obtained colonies were fixed with 25% glutaraldehyde for 20 min and then stained with 0.1% crystal violet for 15 min. The ability to suppress cell proliferation was analyzed with 100% control values that did not treat the compound. 

### 2.5. SK Activity Assay 

SK 1/2 activity was determined using 10 μM Compounds **2** and **4** and PF-543 using the Adapta^TM^ screening system (Thermo Fisher Scientific system, Waltham, MA, USA). SK1 activity assay used 0.04–0.16 ng SK1 enzyme, 50 μM sphingosine lipid substrate in 32.5 mM HEPES, pH 7.5, 0.005% BRIJ 35, 5 μM MgCl_2_, and 0.5 mM EGTA. SK2 activity assay measured 35–140 ng SK2 enzyme, 50 μM sphingosine lipid substrate in 32.5 mM HEPES, pH 7.5, 1.5 mM MgCl_2_, 0.5 μM EGTA. The data were shown as percentage inhibition of SK enzyme by each compound.

### 2.6. Western Blotting

Western blotting measured changes in apoptosis-related proteins (PARP, BAX, BCL-2, Caspase-3). A549 cells (1 × 10^6^ cells per well in 6-well plates) were incubated for 24 h. Cells were treated with concentrations of the compounds and incubated for 24 h. Then, cells were dissolved on ice for two hours with a lysis buffer containing a mixture of protease and phosphatase inhibitors. The lysate was centrifuged at 12,000× *g* for 15 min at 4 °C, and the supernatant was collected. Protein concentrations were measured using a bicinchoninic acid protein assay kit. Total protein was separated by 8–10% sodium dodecyl sulfate–polyacrylamide gel electrophoresis (SDS-PAGE) and transferred onto polyvinylidene fluoride (PVDF) membranes. After being blocked with 5% skim milk, membranes were probed overnight at 4 °C with primary antibodies against SK1, SK2, PARP, BAX, Bcl-2, Caspase-3, and β-actin. Then, the membranes were incubated with subsequent secondary antibodies at room temperature. Then, the protein expressions were measured by Amersham Imager 680 (GE Healthcare, Leiden, WZ, The Netherlands).

### 2.7. RNA Extraction and qRT-PCR 

RNAs were isolated using the Trizol/chloroform reagent (Invitrogen, Waltham, MA, USA). The cDNA synthesis was performed using the PrimeScript 1st strand cDNA synthesis kit according to the manufacturer’s protocol (Takara Bio, Shiga, Japan). For quantitative analysis of various genes, qRT-PCR was performed using the Bio-Rad CFX384 Touch Real-time PCR detection system (Bio-Rad, Hercules, CA, USA) with SYBR Premix Ex Taq (Takara Bio, Shiga, Japan) for 40 cycles. See Table 1.

### 2.8. The Levels of Sphingosine, Ceramide, and S1P

After seeding 1 × 10^6^ cells/well into a 6-well plate and incubating for 24 h, the synthetic compounds were treated by concentration. Then, cells were collected and freezing and thawing were repeated three to five times, and centrifuged supernatant was used for testing. The levels of sphingosine, ceramide, and S1P were measured according to the proposed manual using each ELISA kit (Mybiosource, Inc., San Diego, CA, USA).

### 2.9. Annexin V-FITC

A549 cells (1 × 10^5^ cells) were seeded in into a 12-well plate. Cells were treated at various concentrations of Compounds **2** and **4** and PF-543 for 24 h. After harvesting the cells, the binding buffer was added and then incubated with annexin-V-FITC and PI at room temperature for 15 min. The stained cells were analyzed with MACS Quant Analyzer 10 Flow Cytometer (Miltenyi Biotec, Bergisch Gladbach, Germany) within 1 h.

### 2.10. Mitochondrial Membrane Potential Measurement

To access the mechanism of cell death, *Mitochondrial Membrane Potential* (MMP) was performed using JC-10 cationic dye. A549 cells (1 × 10^5^ cells) were seeded in into 12-well plates and then treated with each concentration of Compounds **2** and **4** and PF-543 for 24 h. Cells were collected, and 20 μM JC-10 dye prepared in HBSS solution (0.14 M NaCl, 0.005 M KCl, 0.001 M CaCl_2_, 0.005 M MgSO_4_-7H_2_O, 0.0004 M MgSO_4_-6H_2_O, 0.0003 M Na_2_HPO_4_-2H_2_O, 0.0004 M KH_2_PO_4_, 0.006 M *d*-glucose, 0.004 M NaHCO_3_, 0.002 M HEPES, and 0.02% Pluronic^®^ F-127) was added to the cell for 30 min and mixed. The samples were protected from light and measured by using the MACS Quant Analyzer 10 Flow cytometer.

### 2.11. siRNA Transfection

Control small interfering RNA (siControl) was obtained from Bioneer (Daejeon, Korea), while SK1 (sc-44114) and SK2 (sc-39225) siRNA (siSK1 and siSK2) were purchased from Santa Cruz Biotechnology (Dallas, TX, USA). Cells were transfected using Lipofectamine RNAiMAX Reagent (Invitrogen, Carlsbad, CA, USA) according to the manufacturer’s instructions. Transfection efficiency was monitored by Western blot. After 24 h of infection, cytotoxicity was measured using infected cells.

### 2.12. In Vitro Metabolic Stability of PF-543 and Derivatives 

Microsomal incubations were performed three times in 0.1 M potassium phosphate buffer (pH 7.4) in 1.5 mL tube. NADPH-dependent metabolism was evaluated by PF-543, derivatives, or Verapamil (positive control) into pooled liver microsomes of human (HLM), dog (DLM), rat (RLM), and mouse (MLM) in the presence of NADPH regenerating system. The final mixtures contained 0.5 mg/mL HLM, 0.1 M potassium phosphate buffer (pH 7.4), and NADPH regenerating system (10 mM MgCl_2_, 1 mM NADPH). HLM was added and the mixture was preincubated at 37 °C in a shaking incubator at approximately 350 rpm for 5 min under Thermo mixer (Eppendorf., Hamburg, Germany). The reaction was started by adding 1 mM NADPH and was then terminated by adding 40 µL of ice-cold acetonitrile containing 10 µM chlorproamide as an internal standard at 0, 30 min. The incubation mixtures were centrifuged at 15,000× *g* for 5 min at 4 °C. A 2 µL of the supernatant was taken and directly injected into the LC-MS/MS system. The LC-MS/MS system is the Nexera XR HPLC system (Shimadzu Co., Kyoto, Japan) coupled to the TSQ Vantage triple quadrupole mass spectrometer equipped with Xcalibur version 1.1.1 (Thermo Fisher Scientific Inc., Waltham, MA, USA).

### 2.13. Experimental Animals

Five-week-old BALB/c-nu/nu male mice were purchased from Orient Bio Inc. (Seongnam, Gyeonggi-do, Korea). The mice were housed in a climate-controlled room (22 ± 2 °C, 50 ± 10% relative humidity) and specific pathogen-free conditions with a 12 h light/dark cycle and adapted for a week with free access to food and water. Cages, bedding, and drinking water were autoclaved and changed regularly. The cancer cell suspension {3 × 10^7^ cells/ 150 μL phosphate-buffered saline (PBS)} was inoculated subcutaneously into the right flank. Measurable tumors were found 7–8 days after cell inoculation, and tumors grew in 90% of the injection site two weeks later. The mice were randomly divided into test groups (12 mice per group) when the tumor volume reached 100 mm^2^ about two weeks after the inoculation (experimental day 0). The control group (vehicle-treated group) and Compound **4** (5 mg/kg) were administered i.p. 3 times a week. The formulation of Compound **4** was prepared in a mixture of 10% DMSO + 90% PBS. The tumor growth rate and body weights in each of the two groups were measured every three days using a Vernier caliper and a digital weighing balance, respectively. Tumor volume was calculated as *l* × *b*^2^ × 0.52, where *l* is the length and *b* is the width. Four weeks after treatment, blood samples were drawn from the orbital sinus under anesthesia, the mice were sacrificed by cervical dislocation, and the tumors were dissected for further examination.

### 2.14. Immunohistochemical Analysis

The paraffin-embedded tumor specimens were deparaffinized with xylene and a series of different alcohol concentrations, rehydrated, and antigen retrieval was carried out with sodium citrate buffer (0.05 M with PH 6). After blocking with 5% BSA, the slides were incubated overnight at 4 °C with KI67 (Cell Signaling Technology, Danvers, MA, USA), Caspase-3 (Abcam, Cambridge, UK), and S1P antibodies (Echelon Biosciences inc, Salt Lake City, UT). The slides were washed with 1× TBST (TBST with PH 8.4) plus 0.5 percent Triton X-100 (Sigma-Aldrich, St. Louis, MO, USA), then secondary antibodies were added in 1:500 ratios (Invitrogen-Alexa Fluor plus 555, 488, and Alexa FluorTM546), and nuclear staining was performed with Hoechst (Invitrogen, Carlsbad, CA, USA), mounted with a flurosave reagent (EMD Millipore Crop, Billerica, MA, USA). Stain sections were observed and photographed using a confocal microscope (Carl-Zeiss-LSM710NLO and LSM780NLO), and results were analyzed using the Carl-Zeiss ZEN2 software and quantification was done by using the ImageJ software, respectively. 

### 2.15. Serum Analysis

Blood samples were centrifuged at 3000× *g* for 20 min, and levels of ALT, AST, ALP, sphingosine, ceramide, and S1P were measured using a kit. 

### 2.16. Statistical Analysis

All experiments were performed at least three times. All data were presented as the mean ± standard deviation (SD), and data visualization was performed using the GraphPad Prism 5.0 software (GraphPad Software, San Diego, CA, USA). Statistical comparison was performed using one-way analysis of variance (ANOVA) followed by Turkey’s multiple comparisons test. A value of *p* < 0.05 was considered statistically significant. 

### 2.17. Chemistry

#### 2.17.1. Materials 

Commercially available reagents were used for the reaction. Column chromatography was performed using silica gel grade 60 (230–400 mesh). All solvents used for the reaction were commercially available anhydrous solvents. The ^1^H-NMR and ^13^C-NMR were measured using JEOL ECZ500R (JEOL Co., Tokyo, Japan), and deuterated solvents at 500 and 125 MHz, respectively. An Agilent Technologies G6520A Q-TOF mass spectrometer (Santa Clara, CA, USA) instrument using electrospray ionization (ESI) was used to measure high-resolution mass spectra.

#### 2.17.2. Synthesis and Characterization of Compounds

##### (*R*)-(1-(4-(Bis(3-methyl-5-((phenylsulfonyl)methyl)benzyl)amino)phenethyl)pyrrolidin-2-yl)methanol (**1**)

To a solution of **8** (50 mg, 0.07 mmol) in acetonitrile (4 mL) was added (*R*)-(-)-prolinol (61 mg, 0.21 mmol). The reaction mixture was stirred at 50 °C for overnight. The mixture was concentrated under reduced pressure and purified by column chromatography on silica gel (CH_2_Cl_2_:MeOH = 5:1) to give Compound **1** (31 mg, 61%): ^1^H NMR (500 MHz, CDCl_3_) δ 7.65–7.61 (m, 4H), 7.61–7.56 (m, 2H), 7.46–7.41 (m, 4H), 6.98 (d, *J* = 8.7 Hz, 2H), 6.94 (s, 2H), 6.79 (s, 2H), 6.71 (s, 2H), 6.51 (d, *J* = 8.8 Hz, 2H), 4.34 (s, 4H), 4.22 (s, 4H), 3.92–3.80 (m, 1H), 3.79–3.71 (m, 2H), 3.70–3.63 (m, 4H), 3.61–3.53 (m, 1H), 3.50–3.40 (m, 1H), 2.22 (s, 6H), 2.07–1.80 (m, 4H); ^13^C NMR (125 MHz, CDCl_3_) δ 148.0, 139.0 (2C), 138.9 (2C), 138.2 (2C), 133.8 (2C), 130.5 (4C), 129.6 (2C), 129.0 (4C), 128.7 (4C), 128.3 (2C), 127.9, 126.4 (2C), 113.1 (2C), 72.3, 67.5, 62.8 (2C), 58.4 (2C), 54.2, 49.1, 38.2, 30.0, 22.8, 21.4 (2C); ESI-HRMS (M + H)^+^ *m/z* calcd for C_43_H_49_N_2_O_5_S_2_ 737.3083, found 737.3098. 

##### 1-(4-(Bis(3-methyl-5-((phenylsulfonyl)methyl)benzyl)amino)phenethyl)piperidin-4-ol (**2**)

To a solution of **8** (50 mg, 0.07 mmol) in acetonitrile (4 mL) was added 4-hydroxypiperidine (61 mg, 0.21 mmol). The reaction mixture was stirred at 50 °C for overnight. The mixture was concentrated under reduced pressure and purified by column chromatography on silica gel (CH_2_Cl_2_:MeOH = 5:1) to give Compound **2** (35 mg, 68%): ^1^H NMR (500 MHz, CDCl_3_) δ 7.65–7.56 (m, 6H), 7.46–7.37 (m, 4H), 6.99 (d, *J* = 8.7 Hz, 2H), 6.94 (s, 2H), 6.78 (s, 2H), 6.72 (s, 2H), 6.52 (d, *J* = 8.7 Hz, 2H), 4.35 (s, 4H), 4.22 (s, 4H), 4.13–4.03 (m, 1H), 3.26–3.02 (m, 4H), 3.10–2.99 (m, 4H), 2.42–2.29 (m, 2H), 2.22 (s, 6H), 1.96–1.80 (m, 2H); ^13^C NMR (125 MHz, CDCl_3_) δ 148.1, 139.0 (2C), 138.9 (2C), 138.2 (2C), 133.9 (2C), 130.5 (4C), 129.6 (2C), 129.0 (4C), 128.7 (4C), 128.3 (2C), 127.9, 126.3 (2C), 113.1 (2C), 67.3, 62.8 (2C), 54.2 (2C), 50.9, 47.0 (2C), 34.6, 33.4 (2C), 21.3 (2C); ESI-HRMS (M + H)^+^ *m*/*z* calcd for C_43_H_49_N_2_O_5_S_2_ 737.3083, found 737.3043.

##### (*S*)-1-(4-(Bis(3-methyl-5-((phenylsulfonyl)methyl)benzyl)amino)phenethyl)pyrrolidin-3-ol (**3**)

To a solution of **8** (50 mg, 0.07 mmol) in acetonitrile (4 mL) was added (*S*)-(-)-3-hydroxypyrrolidine (61 mg, 0.21 mmol). The reaction mixture was stirred at 50 °C for overnight. The mixture was concentrated under reduced pressure and purified by column chromatography on silica gel (CH_2_Cl_2_:MeOH = 5:1) to give Compound **3** (29 mg, 57%): ^1^H NMR (500 MHz, CDCl_3_) δ 7.65–7.61 (m, 4H), 7.61–7.57 (m, 2H), 7.46–7.41 (m, 4H), 6.99 (d, *J* = 8.7 Hz, 2H), 6.94 (s, 2H), 6.76 (s, 2H), 6.74 (s, 2H), 6.52 (d, *J* = 8.8 Hz, 2H), 4.54–4.45 (m, 1H), 4.37 (s, 4H), 4.22 (s, 4H), 3.69–3.61 (m, 1H), 3.53 (dd, *J* = 11.7, 7.2 Hz, 4H), 3.50–3.42 (m, 1H), 3.24 (dd, *J* = 13.9, 6.3 Hz, 1H), 3.07–2.99 (m, 1H), 2.21 (s, 6H), 1.97 (dddd, *J* = 17.1, 9.2, 6.5, 3.5 Hz, 2H); ^13^C NMR (125 MHz, CDCl_3_) δ 148.1, 139.0 (2C), 138.9 (2C), 138.2 (2C), 133.9 (2C), 130.5 (4C), 129.6 (2C), 129.5 (4C), 128.9 (4C), 128.3 (2C), 127.9, 126.3 (2C), 113.1 (2C), 71.1, 69.8, 62.8 (2C), 55.7 (2C), 54.1, 52.3, 41.5, 34.3, 21.4 (2C); ESI-HRMS (M + H)^+^ *m/z* calcd for C_42_H_47_N_2_O_5_S_2_ 723.2926, found 723.2964.

##### (1-(4-(Bis(3-methyl-5-((phenylsulfonyl)methyl)benzyl)amino)phenethyl)piperidin-4-yl)methanol (**4**)

To a solution of **8** (50 mg, 0.07 mmol) in acetonitrile (4 mL) was added 4-piperidine methanol (61 mg, 0.21 mmol). The reaction mixture was stirred at 50 °C for overnight. The mixture was concentrated under reduced pressure and purified by column chromatography on silica gel (CH_2_Cl_2_:MeOH = 5:1) to give Compound **4** (38 mg, 73%): ^1^H NMR (500 MHz, CDCl_3_) δ 7.65–7.64 (m, 4H), 7.60–7.58 (m, 2H), 7.45–7.41 (m, 4H), 6.99 (d, *J* = 8.5 Hz, 2H). 6.97 (s, 2H), 6.84 (s, 2H), 6.70 (s, 2H), 6.50 (d, *J* = 8.6 Hz, 2H), 4.34 (s, 4H), 4.24 (s, 4H), 3.50 (d, *J* = 6.5 Hz, 2H), 3.07 (d, *J* = 11.0 Hz, 2H), 2.76–2.73 (m, 2H), 2.60–2.57 (m, 2H), 2.25 (s, 6H), 2.07–2.03 (m, 2H), 1.78 (d, *J* = 12.0 Hz, 2H), 1.57–1.48 (m, 1H), 1.40–1.33 (m, 2H); ^13^C NMR (125 MHz, CDCl_3_) δ 147.4, 139.2 (2C), 138.8 (2C), 138.1 (2C), 133.7 (2C), 130.4 (4C), 129.4 (4C), 128.9 (4C), 128.7 (2C), 128.3 (2C), 127.9, 126.3 (2C), 112.8 (2C), 67.8, 62.8 (2C), 61.0 (2C), 54.1, 53.5 (2C), 38.4, 32.4, 28.6 (2C), 21.3 (2C); ESI-HRMS (M + H)^+^ *m/z* calcd for C_44_H_51_N_2_O_5_S_2_ 751.3239, found 751.3265.

##### 1,3-Bis(bromomethyl)-5-methylbenzene (**5**)

Mesitylene (3.0 g, 0.025 mol) was put in a sealed tube, dissolved in ethyl acetate (60 mL), *N*-bromosuccinimide (8.88 g, 0.05 mol) was added, and the mixture was stirred at 60 °C for 3 days. The reaction was terminated with water and EtOAc (ethyl acetate). The organic layer was dried over MgSO_4_ and concentrated under reduced pressure. Column chromatography (*n*-hexane:EtOAc = 20:1) was performed to obtain Compound **5** (2.2 g, 32%) from the resulting mixture: ^1^H NMR (500 MHz, CDCl_3_) δ 7.21 (s, 1H), 7.14 (s, 2H), 4.44 (s, 4H), 2.34 (s, 3H); ^13^C NMR (125 MHz, CDCl_3_) δ 139.2, 138.3 (2C), 129.9 (2C), 126.7, 33.0 (2C), 21.2; ESI-HRMS (M + H)^+^ *m/z* calcd for C_9_H_11_Br_2_ 276.9228, found 276.9217.

##### 1-(Bromomethyl)-3-methyl-5-((phenylsulfonyl)methyl)benzene (**6**)

Compound **5** (1.0 g, 0.0036 mol) was put in a sealed tube, dissolved in THF/DMF (tetrahydrofuran/*N*,*N*-dimethylformamide, 2/1, 60 mL), benzene sulfinic acid sodium salt (649 mg, 0.0036 mol) was added, and the reaction was stirred at 80 °C for 3 days. After cooling the reactant to room temperature, the reaction was terminated with water, the organic layer was extracted with EtOAc, dried over MgSO_4_, and concentrated under reduced pressure. Column chromatography (*n*-hexane:EtOAc = 2:1) was performed to obtain Compound **6** (623 mg, 51%) from the resulting mixture: ^1^H NMR (500 MHz, CDCl_3_) δ 7.64–7.59 (m, 3H), 7.48–7.45 (m, 2H), 7.14 (s, 1H), 6.88 (s, 1H), 6.79 (s, 1H), 4.32 (s, 2H), 4.24 (s, 2H), 2.26 (s, 3H); ^13^C NMR (125 MHz, CDCl_3_) δ 139.2, 138.1, 137.7, 133.9, 131.9, 130.3, 129.1 (2C), 128.7 (2C), 128.6 (2C), 62.7, 33.0, 21.2; ESI-HRMS (M + H)^+^ *m/z* calcd for C_15_H_16_BrO_2_S 339.0054, found 339.0021.

##### 2-(4-(Bis(3-methyl-5-((phenylsulfonyl)methyl)benzyl)amino)phenyl)ethanol (**7**)

The 4-aminophenethyl alcohol (500 mg, 3.64 mmol) was dissolved in THF (30 mL), and K_2_CO_3_ (2.0 g, 0.014 mol) and Compound **6** (3.1 g, 0.009 mol) were added thereto. The reaction was stirred at room temperature for 12 h, quenched with water, the organic layer was extracted with EtOAc, dried over MgSO_4_, and concentrated under reduced pressure. Column chromatography (CH_2_Cl_2_:MeOH = 10:1) was performed to obtain Compound **7** (1.02 g, 43%) from the resulting mixture: ^1^H NMR (500 MHz) δ 7.64 (dd, *J* = 8.2, 1.0 Hz, 4H), 7.61–7.56 (m, 2H), 7.45–7.40 (m, 4H), 7.00 (d, *J* = 8.6 Hz, 2H), 6.97 (s, 2H), 6.82 (s, 2H), 6.71 (s, 2H), 6.53 (d, *J* = 8.5 Hz, 2H), 4.35 (s, 4H), 4.23 (s, 4H), 3.78 (t, *J* = 6.4 Hz, 2H), 2.74 (t, *J* = 6.4 Hz, 2H), 2.24 (s, 6H); ^13^C NMR (125 MHz, CDCl_3_) δ 147.7, 139.2, 138.9 (2C), 138.2 (2C), 133.8 (2C), 130.5 (2C), 129.9 (2C), 129.0 (4C), 128.7 (4C), 128.3 (4C), 127.9 (2C), 126.4 (2C), 113.0 (2C), 63.9 (2C), 62.8, 54.2 (2C), 38.2, 21.4 (2C); ESI-HRMS (M + H)^+^ *m/z* calcd for C_38_H_40_NO_5_S_2_ 654.2348, found 654.2316. 

##### 4-(2-Bromoethyl)-*N*,*N*-bis(3-methyl-5-((phenylsulfonyl)methyl)benzyl)aniline (**8**)

CBr_4_ (458 mg, 1.38 mmol) was added into a stirring of Compound of **7** (300 mg, 0.46 mmol) and PPh_3_ (362 mg, 1.38 mmol) in CH_2_Cl_2_ (30 mL), and the reaction mixture was stirred at rt for 12 h. After that, water was added into the mixture and extracted with EtOAc and concentrated under reduced pressure to afford a crude product, which was purified by column chromatography on silica gel (CH_2_Cl_2_:MeOH = 20:1) to give Compound **8** (234 mg, 71%): ^1^H NMR (500 MHz, CDCl_3_) δ 7.66–7.58 (m, 6H), 7.89–7.42 (m, 4H), 7.00 (d, *J* = 6.8 Hz, 2H), 6.97 (s, 2H), 6.85 (s, 2H), 6.71 (s, 2H), 6.53 (d, *J* = 6.8 Hz, 2H), 4.35 (s, 4H), 4.25 (s, 4H), 3.67 (t, *J* = 6.0 Hz, 2H), 2.95 (t, *J* = 6.0 Hz, 2H), 2.25 (s, 6H); ^13^C NMR (125 MHz, CDCl_3_) δ 147.9, 139.0, 138.8 (2C), 138.2 (2C), 133.7 (2C), 130.5 (2C), 129.6 (2C), 129.1 (4C), 128.9 (4C), 128.3 (4C), 127.9 (2C), 126.3 (2C), 112.7 (2C), 62.7 (2C), 54.1 (2C), 45.5, 38.3, 21.3 (2C); ESI-HRMS (M + H)^+^ *m/z* calcd for C_38_H_39_BrNO_4_S_2_ 716.1504, found 716.1563. 

## 3. Results

### 3.1. Synthesis of PF-543 Derivatives

This study attempted to modify the structure of PF-543 to increase its anticancer activity. To maintain the high inhibitory effect of PF-543 on SK1 activity, we tried to develop derivatives that maintained the structure of PF-543 to the maximum extent possible. We simply introduced two similar functional groups centered on amine for the synthesis of new PF-543 analogs with a bulky backbone. We investigated how this large structural change of the tail of PF-543 affects the SK1/2 inhibitory effect and anticancer activity.

**Scheme 1:**
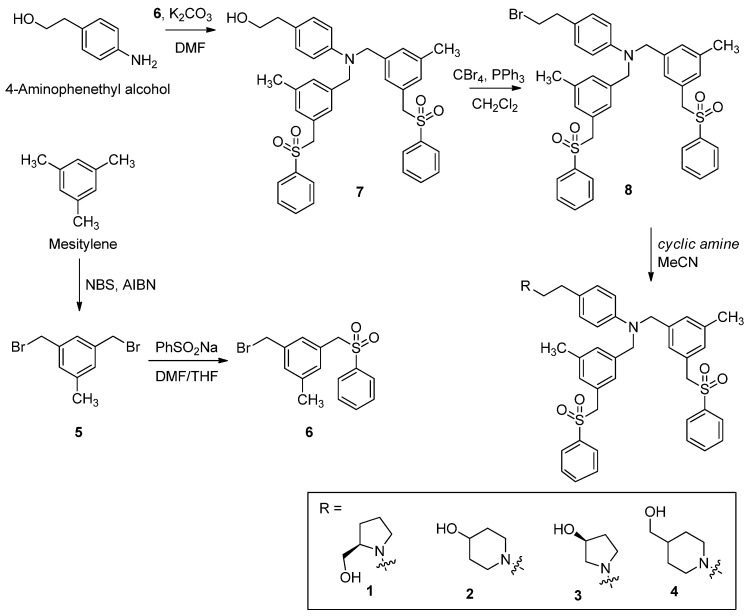
Synthesis of PF-543 derivatives (**1**–**4**).

Compound **5** was selectively brominated at two benzylic positions from mesitylene using *N*-bromosuccinimide (NBS) and Compound **6** was synthesized by introducing a benzenesulfonyl group into Compound **5** in the same manner as PF-543. For the synthesis of Compound **7**, considering the bulky tail group, 4-aminophenethyl alcohol containing one more carbon than PF-543 was used as a starting material for the fluidity of the compound. Potassium carbonate was used as a base to combine Compound **6** and 4-aminophenethyl alcohol, and Compound **7** was brominated using the Appel reaction. Compounds **1**–**4** were finally synthesized by introducing (*R*)-(-)-prolinol, 4-hydroxypiperidine, (*S*)-(-)-3-hydroxypyrrolidine, and 4-piperidinemethanol into Compound **8**, respectively (Scheme 1).

### 3.2. Compounds **2** and **4** Reduce the Viability of Lung Cancer Cell Lines Compared to PF-543

**Figure 2:**
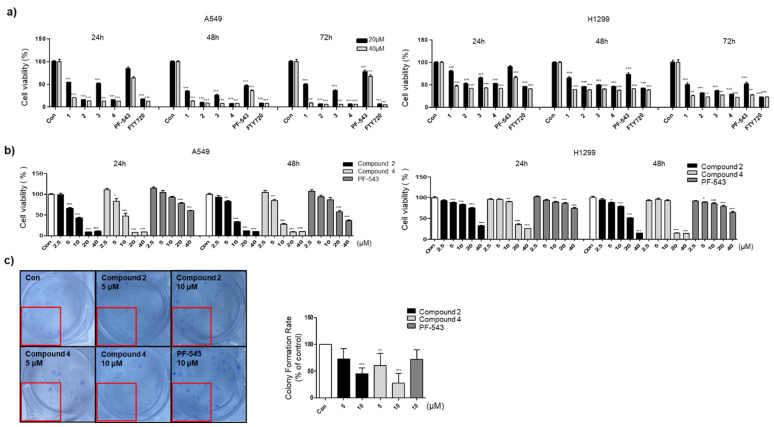
Cytotoxic effects of PF-543 and PF-543 derivatives in lung cancer cells; (**a**) Cell viability treated Compound **1**–**4**, PF-543, and FTY720 for 24, 48, and 72 h in A549 and H1299 cells; (**b**) Compounds **2** and **4** and PF-543 were treated for 24 and 48 h by concentration (2.5, 5, 10, 20, 40 μM) in A549 and H1299 cells; (**c**) Colony formation assay in A549 cells treated with PF-543 and PF-543 derivatives for 24 h. All experiments were repeated three times, and each value is expressed as the mean ± SD (*n* = 3). * *p* < 0.05, ** *p* < 0.01, and *** *p* < 0.001 compared with control group.

We compared and analyzed the cytotoxic effects of synthesized PF-543 analogs **1**–**4**, PF-543, and FTY720 in lung cancer cell lines, A549, and H1299 (Figure 2). From the results measured after 24, 48, and 72 h, Compounds **1**–**4** showed higher cytotoxic effects than PF-543, and Compounds **2** and **4** at 20 and 40 μM concentrations, which have a piperidine head group, showed cytotoxic effects similar to those of FTY720 (Figure 2a). Based on the analysis of the cytotoxic effects of Compounds **2** and **4** in lung cancer cells after 24 h and 48 h, it was identified that Compounds **2** and **4** showed IC_50_ = 7.07 and 7.75 μM, respectively, after 24 h, and similar cytotoxic effects were observed after 48 h (Figure 2b). A colony formation assay was performed to observe whether the compounds were involved in the inhibition of proliferation of lung cancer cells (Figure 2c). A549 cells were treated with PF-543 derivatives **2** and **4** at 5 and 10 μM, and colonies were effectively reduced in the derivative-treated group than in PF-543 treated group. It has also been shown that Compounds **2** and **4** reduce colony formation in a concentration-dependent manner. Based on these results, it was observed that Compounds **2** and **4** were more effective as anticancer agents than PF-543.

### 3.3. Compounds **2** and **4** Reduce S1P Formation and Increase Ceramide Levels in A549 Cells 

**Figure 3:**
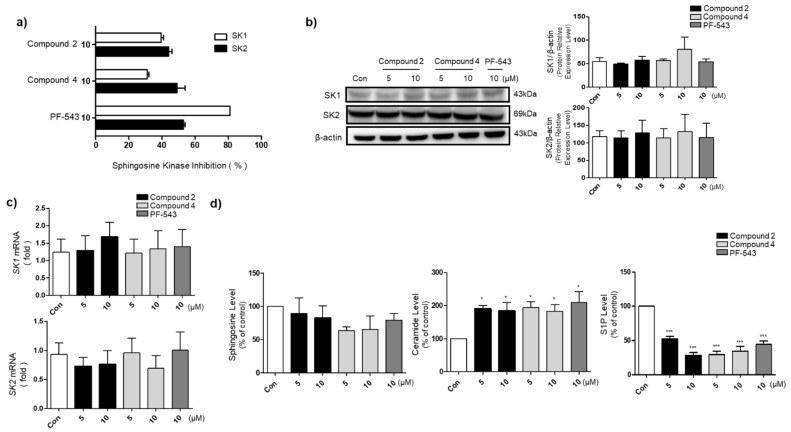
Effects of PF-543 and PF-543 derivatives on A549 cells biological result; (**a**) Activities of SK1 and SK2 at 10 μM concentrations of PF-543 and PF-543 derivatives; (**b**) Protein was extracted and detected SK1, SK2, and β-actin protein levels by Western blotting. Each protein level was normalized by β-actin. Western blot was performed in three independent experiments; (**c**) The mRNA levels of *SK1* and *SK2* were quantified by qRT-PCR by treating A549 cells with PF-543 or PF-543 derivatives at 5 and 10 μM for 24 h. SK1 and SK2 mRNA was normalized to cyclophilin; (**d**) Sphingosine, ceramide, and S1P levels were measured in A549 cells with 5 and 10 μM PF-543 and PF-543 derivatives. The data are expressed as the mean ± S.D. * *p* < 0.05, *** *p* < 0.001 compared with control group.

Since Compounds **2** and **4** are the derivatives of PF-543, their inhibitory effect on SK1/2 was compared with PF-543 at a concentration of 10 μM (Figure 3a). Compounds **2** and **4** exhibited 39 and 31% of SK1 inhibitory efficacy and 44 and 49% of SK2 inhibitory efficacy, respectively, at a concentration of 10 μM. Compounds **2** and **4** demonstrated no improvement in the SK1 inhibitory activity compared with PF-543, and PF-543 had a maximum inhibitory effect on SK1. Both the PF-543 analog and PF-543 showed similar SK2 inhibitory ability. The lower selectivity for SK1 compared to PF-543 appears to be affected by the analog’s bulky tail. Protein and mRNA levels were analyzed to determine whether the synthesized compound modulates the downstream signaling pathway through the regulation of SK1 and SK2 expression. Since Compounds **2** and **4** did not affect the expression of proteins and mRNAs of SK1 and SK2, it could be predicted that they exhibited physiological activity mediated by activating SK1 and SK2 rather than affecting their expression (Figure 3b,c). The ceramide levels, which indicate the intensity of apoptosis, were increased by treatment with Compounds **2** and **4**. The levels of S1P, which is produced by SK and promotes the growth of cancer cells, were reduced by treatment with Compounds **2** and **4** (Figure 3d).

### 3.4. Compounds **2** and **4** Induce Apoptosis through Mitochondrial Membrane Potential Depolarization and Active Intrinsic Apoptosis Pathway

**Figure 4:**
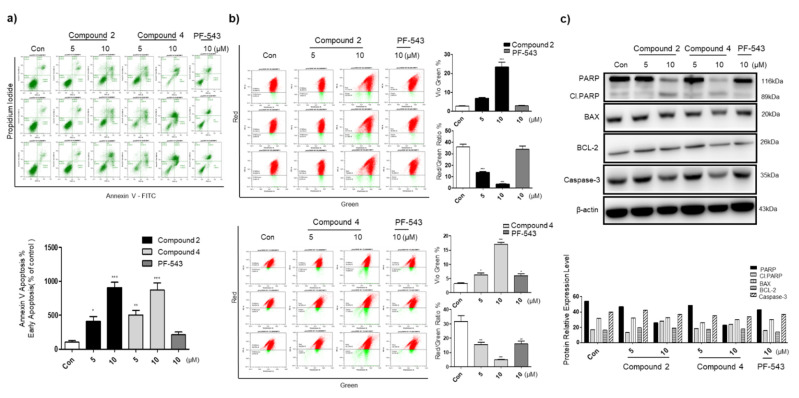
Effects of Compounds **2** and **4** on apoptosis and mitochondrial membrane potential in A549 cells; (**a**) A549 cells were treated with Compounds **2**, **4** and PF-543 of the desired concentration, and apoptosis analysis was performed with annexin V-FITC assay; (**b**) The mitochondrial outer membrane potential was measured using a JC-10 dye and analyzed by the flow cytometer; (**c**) The expression of apoptosis pathway proteins was observed after 24 h treatment with PF-543 and PF-543 derivatives at concentrations of 5 and 10 µM in A549 cells. β-actin was used as the loading control. Western blots are representative of three independent experiments. Data are expressed as the mean ± S.D. * *p* < 0.05, ** *p* < 0.01, and *** *p* < 0.001 compared with control group.

To confirm the apoptotic effect of Compounds **2** and **4**, the level of annexin-V was measured in lung cancer cell line A549 at concentrations of 5 and 10 μM (Figure 4a). Compounds **2** and **4** showed a significantly higher apoptotic effect compared to PF-543 at 10 μM, and a significant effect was apparent even at 5 μM. As a result of measuring the change in mitochondrial membrane potential (MMP) using JC-10, it was found that when cells were treated with Compounds **2** and **4** at 10 μM (Figure 4b), the MMP was significantly increased compared to that after treatment with PF-543. The results of annexin V and JC-10 revealed that cell apoptosis in the presence of Compounds **2** and **4** was higher and concentration-dependent than that in the case of PF-543. 

We tried to observe changes in the activity of prosurvival proteins and proapoptotic proteins in the cell line in the presence of PF-543 and derivatives (Figure 4c). Western blotting analysis revealed that the levels of PARP and Caspase-3 were decreased and cleavage of PARP was increased in the Compounds **2** and **4** treatment group. Based on these results, it was confirmed that PF-543 derivatives induce apoptosis by regulating the levels of apoptosis-related protein expression in A549 lung cancer cells. 

### 3.5. Compounds **2** and **4** Selectively Act on SK1 to Induce Cytotoxicity

**Figure 5:**
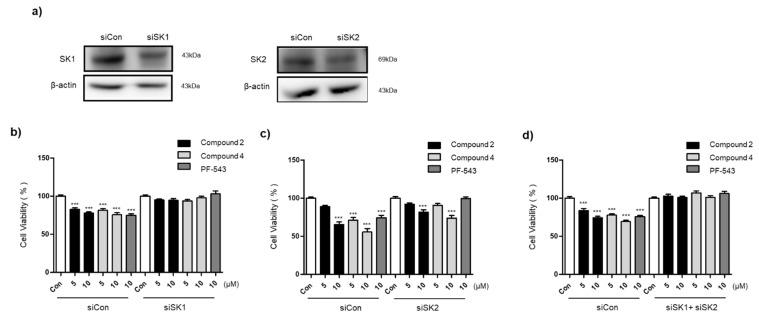
Cell cytotoxic effect of Compounds **2** and **4** by inhibiting SK1 and SK2 expression. A549 cells were transiently transfected with control siRNA or SK1, SK2 siRNA for 24 h; (**a**) The protein expression level of SK1 and SK2 was confirmed by Western blot; (**b**–**d**) Cells transfected with siSK1, siSK2 or siSK1 + siSK2 were treated with Compounds **2** and **4** and FP-543 for 24 h, respectively, and cell viability was measured using EZ-CYTOX. Data are expressed as the mean ± S.D. *** *p* < 0.001 compared with control group.

To confirm whether the lung cancer cell cytotoxicity in the presence of the compounds was mediated through SK1 and SK2, the efficacy of the compound was confirmed after knockdown (KO) of SK1 and SK2 using an intracellular siRNA system (Figure 5). Western blot analysis was performed to determine if the expression of SK was reduced using siRNA, and it was found that the expression levels of SK1 and SK2 proteins were decreased by siRNA transfection compared to siControl (Figure 5a). After transfection with siSK1, the MTT analysis revealed that the cytotoxicity of the synthetic compounds was reduced compared to that of siControl (Figure 5b). These results suggest that the compounds exhibit SK1-dependent cytotoxic and apoptotic effects. As a result of examining the efficacy of the compound after SK2 inhibition, it was revealed that cytotoxicity was reduced compared to siControl, but the effect was not offset compared to that of SK1. Thus, cytotoxic efficacies of Compounds **2** and **4** are more dependent on SK1, although SK2 is also partially involved.

### 3.6. The Bulky Tail Structure of Compounds **2** and **4** Increases MS

We have reported that the MS of PF-543 is poor in our previous studies [12]. The stability of synthesized Compounds **2** and **4** was measured to confirm whether the bulky tail structure increases the stability. The stability of all three compounds was confirmed in human, dog, rat, and mouse models, and the reproducibility of the experiment was confirmed using verapamil as a reference drug (Table 2). Both Compounds **2** and **4** showed an increase in MS compared to PF-543. These results show that the tail group transformation of PF-543 is an important aspect in improving the MS of the compound. 

### 3.7. Compound **4** Induces Growth Inhibition and Apoptosis of A549 Cells in BALB/c Nude Mice Tumor Xenograft

**Figure 6:**
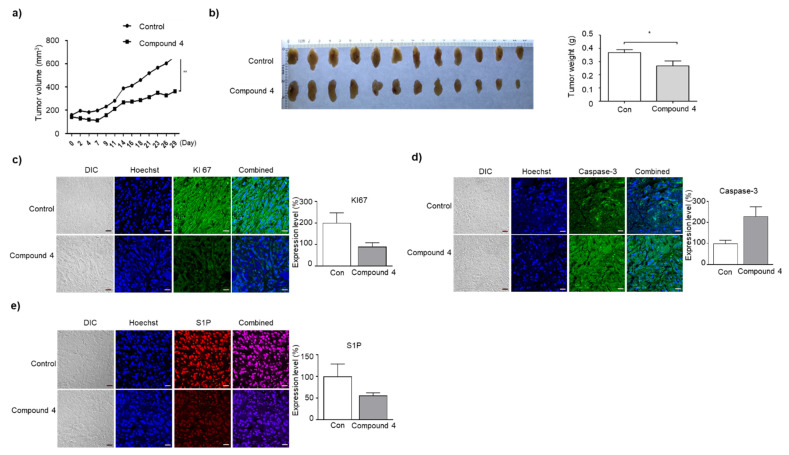
Compound **4** inhibits the growth of A549 cell-induced tumor xenograft in nude Balb/c mice; (**a**) The effectiveness of inhibiting tumor growth was observed in A549 tumor-bearing nude Balb/c mice after intraperitoneal administration of vehicle or Compound **4** (5 mg/kg) 3 times a week for a period of 29 days; (**b**) Photographs were taken and weights were measured for isolated tumors from each group on day 29 after treatment; (**c**–**e**) Immunohistochemistry of tumor sections was performed for KI67, caspase-3 and S1P (scale bar 10 μm, magnification 200X). Data are expressed as the mean ± S.D. * *p* < 0.05, and ** *p* < 0.01, compared with control group.

Among the two compounds, we selected Compound **4** with improved MS and observed its efficacy in xenograft models using lung cancer cell lines, A549. In an experiment conducted for 29 days after treatment with Compound **4**, a statistically significant inhibitory effect on the tumor volume was observed compared to the control group (Figure 6a). A significant decrease in the tumor weight was observed in the Compound **4** treatment group compared to the control group (Figure 6b). There was no difference in overall survival between the two groups, as the mice did not die in both the control and Compound **4**-treated groups during the 4 weeks of drug administration (Supplementary Figure S2). There was no difference in the weight of each group of animals. KI67 staining, a proliferation marker, results demonstrated a decrease in the level of KI67 in the group treated with Compound **4** compared with the control group (Figure 6c). It was found that the level of caspase-3, an apoptosis marker, was increased in the Compound **4** group compared to the control group (Figure 6d). S1P levels in the tumor section were decreased in the Compound **4** treated group (Figure 6e). Therefore, it can be seen that the effect of Compound **4** represents the anticancer effect based on the reduction in S1P mediated by SK inhibition. To examine whether treatment with Compound **4** caused hepatotoxicity, we measured serum biochemical assays. After Compound **4** treatment, there was no change in the levels of ALT and AST enzymes and a significant decrease in the levels of ALP enzyme was observed. Treatment with Compound **4** does not appear to cause hepatotoxicity compared to the control group, and since ALP is also an indicator of the condition of not only the liver, but also other organs such as bones, the significance of the reduction by Compound 4 needs further study. The analysis of S1P, ceramide, and sphingosine in the serum revealed no changes in the amount of sphingolipid in response to treatment with Compound **4** (Table 3).

## 4. Discussion

The production of S1P is regulated by SK1 and SK2. S1P produced by SK1 functions through the G protein, and overexpression of SK1 and the resulting increase in S1P promotes the growth and survival of cancer cells [3]. According to previous reports, the expression levels of SK1 and SK2 are known to increase in patients with NSCLC [16,17], but data on SK and its product S1P in NSCLC are limited. Currently, the SK2 inhibitor, ABC294640 is in the stage of clinical trials as a treatment for cancers such as cholangiocarcinoma and prostate cancer. As such, SK inhibitors have the advantage that they can be developed as various cancer treatments, but no additional compounds have been reported that have entered clinical trials even though many inhibitors have been developed so far. The relationship between the selectivity of SK1/2 and the anticancer activity is not clear to date, and the low anticancer activity of PF-543, which has a particularly high SK1 inhibitory effect, makes this study difficult. In addition, the low MS of PF-543 requires structural improvement to achieve stability. Although PF-543 effectively reduced the production of S1P, the reason why it failed to induce apoptosis of cancer cells is still unclear. It is also possible that PF-543 exhibits apoptosis deficiency through inhibition of other enzymes in the sphingolipid pathway that rebalances the level of ceramides [4].

We synthesized four novel PF-543 analogs by introducing two tails with the same structure as PF-543 centered on nitrogen. The synthesized compounds were analyzed for anticancer activity in lung cancer cells, and the piperidine form (Compounds **2** and **4**) showed better anticancer activity than the pyrrolidine form (Compounds **1** and **3**) in the head group. Compounds **2** and **4** having a piperidine head group showed lower selectivity for SK1 compared to PF-543. Compound **4** inhibited S1P at a level similar to that of PF-543 and induced a similar level of ceramide production. Compound **4** induced slightly lower sphingosine expression than PF-543, but Compound **2** induced sphingosine expression at a level similar to that of PF-543. Therefore, the low anticancer activity of PF-543 is still difficult to be interpreted based on sphingolipid analysis. 

EGFR is overexpressed in patients with NSCLC and its expression is associated with poor prognosis. EGFR is a membrane protein that regulates key aspects of cell proliferation, angiogenesis, and metastasis [18]. The EGFR activating mutation consists of the deletion mutation in exon 19 and the L858R mutation in exon 21 [19]. The A549 and H1299 cell lines that were used in the experiment are EGFR wild-type, and the HCC827 cell line shown in Supplementary Figure S1, consists of EGFR exon 19 deletion mutation. We tried to find out the changes in our synthetic compounds according to cells with or without EGFR mutations. The new synthetic compound showed similar cytotoxicity compared to erlotinib, a targeted treatment used in exon 21 deletion mutant patients. Therefore, our new synthetic compound and its relationship to EGFR should be further studied in futuristic studies.

Compounds **2** and **4** showed improved apoptosis compared to PF-543 based on annexin-V and JC-10 analysis. Although the molecular weight was increased due to the dimeric tail, the MS of both Compounds **2** and **4** was improved compared to that of PF-543. Though there was a small difference in the MS results between Compound **4** and Compound **2**, it seems that the head group also affects the stability. However, the novel dimeric analogs using the structure of PF-543 as it is still showed low MS. As the recently reported PF-543 analogs of the triazole tail [20] also showed low MS, it seems necessary to improve the overall structure of PF-543 including the backbone structure. 

Compound **4** showed an anticancer effect in lung cancer animal experiments with increased stability and relatively increased selectivity of the SK2 inhibitory effect compared to PF-543. It is hypothesized that these results will provide important information for the design of SK2 inhibitors, which have been rarely developed compared to SK1, and will help conduct research on whether SK2 inhibitors are advantageous for anticancer activity compared to SK1 inhibitors through comparative studies. Additionally, it is necessary to study whether this dimeric structure is structurally applicable to other sphingolipid modulators, and a close study is needed to determine whether PF-543 acts on any other sphingolipid modulators besides SK.

## Figures and Tables

**Table 1:** Primer sequences of *SK1* and *SK2* genes used in qRT-PCR.

Gene	Primer Sequences
*SK1*	Forward: 5′-GGCGTCATGCATCTGTTCTA-3′
	Reverse: 5′-ACACACCTTTCCCATCCTTG-3′
*SK2*	Forward: 5′-ATCTCTGAAGCTGGGCTGTCC-3′
	Reverse: 5′-CTCCCAGTCAGGGCGATCTA-3′

**Table 2:** Metabolic stability of **2**, **4**, and PF-543.

MS	HLM	DLM	RLM	MLM
Compound **2**	18.1	18.6	29.1	24.6
Compound **4**	30.2	26.1	22.9	38.5
PF-543	6.4	7.3	7.8	9.4
Verapamil	13.4			

**Table 3:** Change of blood chemistry in tumor xenograft mice for 29 days treated with vehicle and Compound **4**.

	Con	Compound 4
ALT	101.61 ± 5.27	93.64 ± 11.28
AST	98.21 ± 6.53	93.44 ± 14.57
ALP	100.52 ± 4.34	79.54 ± 7.08 ***
Sphingosine	4.37 ± 5.39	7.15 ± 6.61
Ceramide	6.87 ± 3.26	6.2 ± 3.24
S1P	10.2 ± 5.49	11.43 ± 5.26

*** *p* < 0.001 compared with control group.

## Data Availability

Not applicable.

## Supplementary Materials

The following supporting information can be downloaded at: https://www.mdpi.com/article/10.3390/pharmaceutics14102035/s1, Figure S1: Cell cytotoxic effect of compound **2** and compound **4** in HCC827 cell line; Figure S2: Overall survival with and without compound 4 treatment in A549 cell-induced tumor xenograft mice.
