# Supplementary material for: Novel Dimer Derivatives of PF-543 as Potential Antitumor Agents for the Treatment of Non-Small Cell Lung Cancer
Source: Pharmaceutics. 2022 Sep 24;14(10):2035. doi: 10.3390/pharmaceutics14102035 (PMC9611471; doi:10.3390/pharmaceutics14102035)
Supplement: Supplementary file 1 [file pharmaceutics-14-02035-s001.zip › pharmaceutics-1884655-supplementary.pdf]

Supplementary figure S1

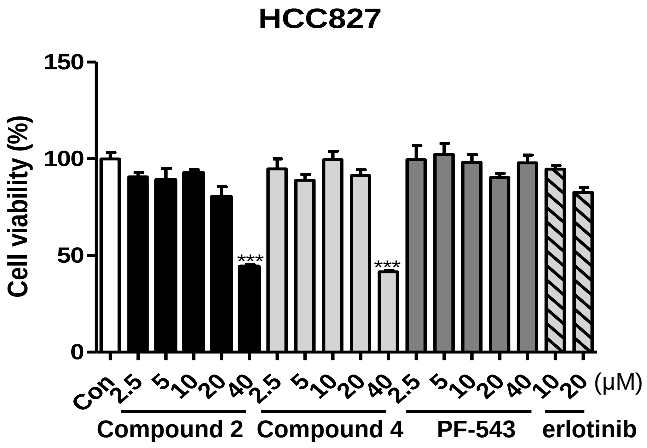

Supplementary figure S1. Cell cytotoxic effect of compound **2** and compound **4** in HCC827 cell line. HCC827 cells were seeded in 96 well plates and treated with 2.5-40 μM of compounds for 24h. Cell viability was assessed using MTT assay. The result is representative of three independent experiments and data are presented as mean ± SD. *** $p < 0.005$ compared with non-treated control group.

Supplementary figure S2

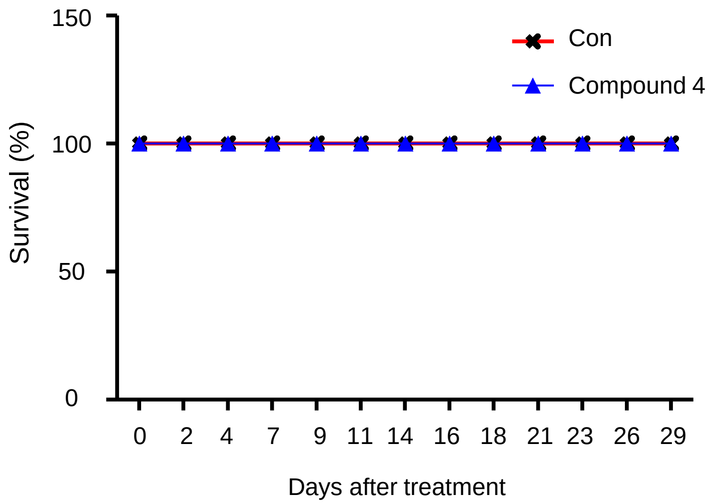

Supplementary figure S2. Overall survival with and without compound **4** treatment in A549 cell-induced tumor xenograft mice. The overall survival was observed for 29 days with intraperitoneal administration of vehicle (n=12) or compound **4** (5 mg/kg, n=12) 3 times a week.
